# Intra-abdominal desmoplastic small round cell tumor: Presentation of four cases and review of the literature

**DOI:** 10.4103/0971-5851.68849

**Published:** 2010

**Authors:** Biswa Mohan Biswal, Venkatesh R. Naik, Syed Ejaz Shamim

**Affiliations:** *Department of Nuclear Medicine, Radiotherapy and Oncology, School of Medical Sciences, Health Campus, Universiti Sains Malaysia, Kelantan, Malaysia*; 1 *Department of Pathology, School of Medical Sciences, Health Campus, Universiti Sains Malaysia, Kelantan, Malaysia*

**Keywords:** *Abdomen*, *chemotherapy*, *desmoplastic small round cell tumor*, *management and prognosis*, *radiotherapy*, *surgery*

## Abstract

Desmoplastic small round cell tumor (DSRCT) of the abdomen is a recently identified aggressive neoplasm. Very few cases have been reported in the literature. Thus, the treatment guidelines are yet to be defined. The role of chemotherapy, radiotherapy and surgery is evolving. We treated four cases of DSRCT involving the abdomen using combination chemotherapy and/or tumor cytoreductive surgery. There were two men and two women. The chemotherapy drugs consisted of cisplatin, adriamycin, etoposide, ifosphamide, vincristine and cyclophsophamide. All patients achieved meaningful partial response to chemotherapy, which maintained for 6–9 months. There were very minimal chemotherapy-related complications. At the time of reporting, the median survival time was 15 months. Thus, DSRCT is an aggressive intra-abdominal tumor with excellent chemoresponsiveness, but relapse is frequent.

## INTRODUCTION

Desmoplastic small round cell tumor (DSRCT) is a recently identified histopathological entity. This malignancy was first described by Gerald and Rosai from Memorial Sloan Kettering Cancer Centre, New York.[1] Since then, about 200 cases have been reported in the English literature as case reports due to its rarity. This malignancy predominantly affects adolescent men in their early twenties. The characteristic presentation of DSRCT is massive abdominal mass and abdominal discomfort, with or without pain. Very rarely, DSRCT can arise from head and neck, base of skull, paratesticular region, ovary, brain and thoracic viscera.[2] After its appearance, it grows very fast in the transcoelomic route and subsequently spreads through the blood stream to the liver, lungs and bones. The cell of origin is not yet known. However, it is postulated to originate from the serosal lining. A specific chromosomal translocation t (11;22) (p13;q12) has been documented in DSRCT.[1] Sometimes, tumor marker CA-125 is raised in DCRCT, involving the pelvic viscera.[3] The biological behavior of the DSRCT is very aggressive, leading to relentless disease progression and low survival record.[4]

Management of DSRCT includes early surgical removal of the tumor followed by high-dose aggressive chemotherapy. Some authors recommended chemotherapy supplemented with stem cell transplantation.[5] Radiotherapy is most often used for palliation of symptomatic disease. However, a recent study by Goodman *et al*. used whole abdominal radiotherapy as a consolidation treatment after chemotherapy in low-volume abdominal disease.[6] In general, most of the patients present with extensive abdominal disease, and they are inoperable to start with. Thus, they are managed with combination aggressive chemotherapy. Here, we would like to present our experience in the multidisciplinary management of DSRCT.

## CASE REPORTS

### Case 1

**Figure 1:**
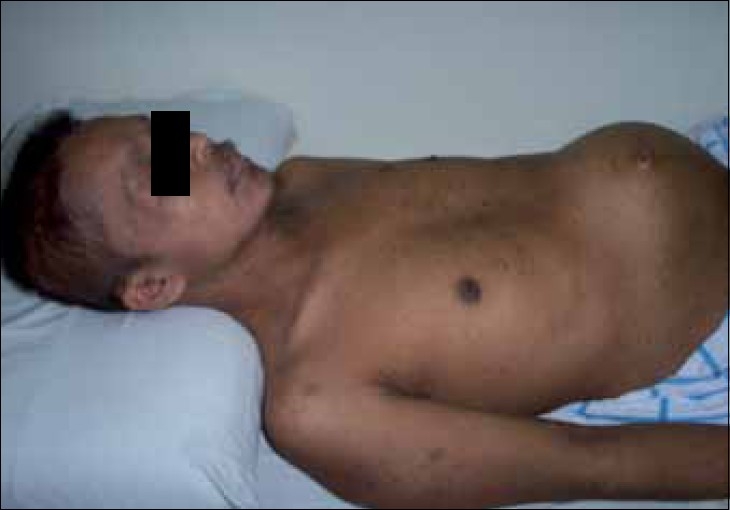
Clinical picture showing extensive intra-abdominal mass

Mr. WRI is a 33-year-old gentleman who presented with complaints of abdominal pain and lump for 2–3 months duration. The pain was ill-defined and confined to the hypochondrium and was associated with distention of the abdomen. There was distended abdomen and dull on percussion. Initially, he was managed with symptomatic medications without any benefit. Subsequently, he was found to have multiple lumps all over the abdomen [Figure 1]. Contrast-enhanced computed tomography (CT) scan of the thorax, abdomen and pelvis revealed multiple enhancing mass lesions arising from the retroperitoneal area. However, the bowel was free. An incisional biopsy was taken and subjected to histopathological evaluation. The microscopical evaluation showed malignant tumor cells arranged in sheets and islands, separated by abundant desmoplastic stroma. The tumor cells are small and round, with hyperchromatic nuclei and scanty cytoplasm Figures 2a–b. There was abundant mitosis. The immunohistochemical stain was positive for EMA, NSE and vimentin. The above features were compatible with DSRCT. In view of the above diagnosis and inoperable nature of the disease, he was advised combination chemotherapy. The chemotherapy regimen consisted of cisplatinum 40 mg/m^2^, ifosphamide 2 g/m^2^ (with mesna rescue), adriamycin 10 mg/m^2^ and etoposide 100 mg/m^2^ for 3 days every 4 weeks for six cycles. However, the patient only received two cycles in view of extreme chemotherapy-induced fatigue. Following the above chemotherapy, the patient achieved complete response and survived without symptoms 12 months postdiagnosis of disease.

**Figure 2a:**
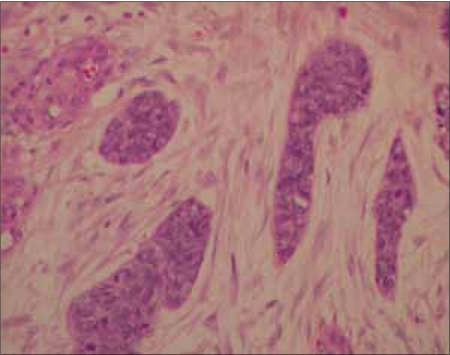
Nests of tumor cells surrounded by desmoplastic stroma, hematoxylin and eosin stain 10× (H&E stain)

**Figure 2b:**
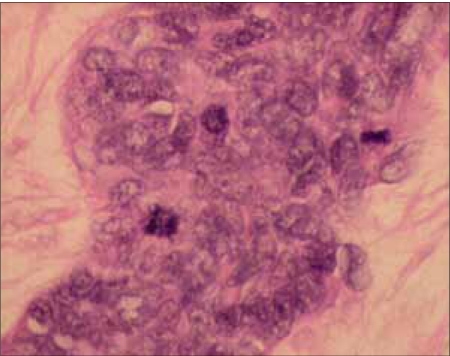
The tumor cells are small, having a hyperchromatic nucleus, and scanty cytoplasm mitotic activity is easily seen, 40×, H&E stain

### Case 2

ZA, a 27-year-old lady, was seen by a surgeon with history of abdominal mass and pain for 1 month duration. The abdominal mass was arising from the pelvis and was fixed to the underlying viscera. A diagnostic CT scan was ordered and she was found to have a huge pelvic mass of size 13.5×13.9×21.9 cm. There were multiple ill-defined hypodense lesions suggestive of liver metastases. There was also a single lung nodule in the upper lobe of the right lungs. The histopathological examination of the biopsy specimen revealed features of DSRCT. In view of metastatic disease, she was advised VAC/IE combination chemotherapy. However, the patient developed progressive disease and succumbed to disease 3 months later.

### Case 3

SZ, a 33-year-old man, was seen by our surgical service with symptoms of abdominal tenderness and altered bowel habit for 1 month duration. There was also associated loss of appetite and loss of body weight. Clinical examination revealed multiple intra-abdominal lumps all over the abdomen. Colonoscopy was performed and was found to be normal. Contrast-enhanced CT scan of the thorax, abdomen and pelvis showed a bizarre contrast-enhanced mass lesion in the intraperitoneal area in the pelvic cavity, possibly invading the small and large bowels. There was presence of lesions in the liver. Excision biopsy specimen was subjected to histopathological evaluation, which showed islands of tumor cells separated by wide brands of fibrous tissue. The tumor cells were small and round, with enlarged nucleus and scanty cytoplasms. The mitotic figures were abundant, with individual cell necrosis. Immunohistochemistry test showed positivity for cytokeratin, NSE and desmin. The stroma was made uniform spindle-shaped embedded in collagen. The other hematological and chemistry parameters were normal. He was offered alternate VAC/IE combination chemotherapy. The chemotherapy consisted of cyclophosphamide 1.2 g/m^2^, vincristine 2 mg and adriamycin 60 mg/m^2^ for 1 day followed by a gap of 3 weeks. Phase two chemotherapy consisted of ifosphamide 1.2 g/m^2^ and etoposide 100 mg/m^2^ for 5 days. The patient received four cycles of the above chemotherapy and achieved good partial response. Subsequent evaluation with CT scan showed good partial response and hence the patient was subjected to surgical debulking operation. Following surgery, the patient noticed epigastric pain and CT scan revealed multiple liver metastases. In view of painful liver metastases, local radiotherapy was delivered to the hepatic areas. A dose of 30 Gy in 15 fractions was delivered using a 6 MV linear accelerator. The patient achieved good palliation and is alive with metastatic disease at the end of 18 months from the date of diagnosis.

### Case 4

A 14-year-old girl presented to her gynecologist with a complaint of suprapubic mass for 2-3 months duration. The lump was gradually increasing in size within a short span. The lump was associated with pain. Initial gynecological ultrasound revealed an ovarian mass. Laparotomy was performed. Peroperatively, the patient presented a solid and cystic omental mass, which was highly vascularized, measuring 22 cm×17 cm×13 cm. CT scan evaluation of the abdomen and pelvis revealed multiple seedlings varying in size all over the peritoneum, including the undersurface of the diaphragm. There was also liver nodularity felt and multiple enlarged nodules adhered and infiltrated into the pelvic sidewalls. The superior part of the tumor was adhered to the greater curvature of the stomach from which a wedge resection was made. The histopathological evaluation of the excised specimen showed sheets and clusters of well-demarcated nests of tumor cells with areas of necrosis and hemorrhage. The cells were small and monomorphic with hyperchromatic nuclei. The cells contain scanty cytoplasm and numerous mitotic figures (17/10 hpf). Occasional rosette formation was also seen. The postoperative period was uneventful and she was referred to the oncologist for further management. The preoperative CA125 tumor marker was 290.9 U/ml. She was treated with CAP chemotherapy regimen to a dose of cyclophosphamide 500 mg/m^2^, adriamycin 50 mg/m^2^ and cisplatinum 50 mg/m^2^ every three weekly for six cycles. Following the above chemotherapy, she became asymptomatic, with very good partial response. On subsequent follow-up, her follow-up CT scan revealed residual disease in the abdomen. She was subsequently offered chemotherapy consisting of ifosphamide and etoposide for four cycles. However, she did not responded and developed progressive abdominal discomfort and abdominal distension due to disease. She subsequently died of disease 21 months post diagnosis.

## DISCUSSION

DSRCT of the abdomen is an enigmatic disease whose biological behavior, genetics and morphological characteristics are yet to be elucidated. In view of nonspecific tumor biological understanding, the management of DSRCT is also not straightforward and specific. Further, formal recommendation of any treatment regimen is handicapped due to the rarity of the disease. The current knowledge about its management is generally based on the anecdotal case reports in the literature and the experience from major referral centers like Memorial Sloan Kettering Cancer Center and Italian cancer centers.[5,7] In general, blanket management policy of management should include tissue diagnosis and detection of DSRCT at an early and operable stage. The initial treatment approach should be primary cytoreductive surgery to achieve minimal tumor load in the abdomen so that the further adjuvant therapy is more effective.[8] However, most patients are inoperable at the time of presentation. Thus, DSRCT cases were managed with neoadjuvant chemotherapy followed by resection of residual primary tumor.[3] All our cases, except case-3, were discovered at an inoperable stage and hence we tried to treat them with the combination chemotherapy regimen.

DSRCT is very responsive to combination chemotherapy.[9] The cytotoxic agents that seem to be effective in DSRCT are cyclophosphamide, ifosphamide, adriamycin, vincristine, etoposide and topotecan.[7] The most famous chemotherapy protocols are the P6 protocol developed by Kushner *et al*. of the Memorial Sloan-Kettering Cancer Centre, New York, USA The above regimen is offered for seven cycles, followed by delayed cytoreductive surgery and consolidated radiotherapy. The European chemotherapy protocols are equieffective, but are prescribed for four cycles, followed by similar management with surgery and radiotherapy.[10] The response to chemotherapy is excellent. Despite excellent chemotherapy response in DSRCT, the long-term survival results are dismal.[4] The disease usually fails in the liver and intraperitoneal seedling due to development of quick chemotherapy resistance. Most chemotherapy protocols are aggressive, with high-dose chemotherapy agents followed by stem cell rescue.[5]

The role of radiotherapy is not consistent throughout the literature. Most studies reported radiotherapy for the palliation of symptomatic metastatic sites or to consolidate the residual disease in the abdomino-pelvic region. Very recently, Goodman *et al*.[6] presented their experience on the use of whole-abdomen radiotherapy (30 Gy) in 21 cases of DSRCT after complete surgery and seven cycles of P6 chemotherapy protocol. Although the complication figures were notable, the authors considered the local control and survival as meaningful as the long-term survival is very short. Therefore, the patient might die before the manifestation of late radiotherapy complications. In our series, we used local radiotherapy to relieve pain due to liver metastasis; however, the pain control and palliation were encouraging.

Survival of DSRCT patients after successful treatment is variable. Very rarely, the patient survives beyond 24 months. The results of combination of optimal cytoreductive surgey followed by multiagent chemotherapy resulted in a 3-year survival rate of 58% compared to inoperable patients.[11] However, other authors found a median survival of 34 months compared to 14 months for operable and inoperable tumors, respectively.[12] The longest survivor after the diagnosis of DSCRT was reported by Gil *et al*. to be 101 months.[13]

In conclusion, in this presentation, we highlight the management of four cases of DSRCT using multimodality treatment. The chemotherapy response was encouraging in all cases, although none of our patients maintained response beyond 6–7 months. In view of the aggressive nature of the disease, patients should be subjected to primary surgery followed by high-dose combination chemotherapy and stem cell rescue. At present, whole-body radiotherapy cannot be recommended due to the risk of bowel complications. Alternatively, radiotherapy should be reserved for palliation of symptomatic disease and to control residual disease after chemotherapy. Further, molecular characterization of DSRCT should be performed in the future to design targeted therapies in this aggressive neoplasm.
